# Changes in the Expression of Genes Regulating the Response to Hypoxia, Inflammation, Cell Cycle, Apoptosis, and Epithelial Barrier Functioning during Colitis-Associated Colorectal Cancer Depend on Individual Hypoxia Tolerance

**DOI:** 10.3390/ijms25147801

**Published:** 2024-07-16

**Authors:** Dzhuliia Dzhalilova, Maria Silina, Ivan Tsvetkov, Anna Kosyreva, Natalia Zolotova, Elena Gantsova, Vladimir Kirillov, Nikolay Fokichev, Olga Makarova

**Affiliations:** 1 Avtsyn Research Institute of Human Morphology of Federal State Budgetary Scientific Institution “Petrovsky National Research Centre of Surgery”, 117418 Moscow, Russia; marusyasilina99@yandex.ru (M.S.); davedm66@gmail.com (I.T.); kosyreva.a@list.ru (A.K.); natashazltv@gmail.com (N.Z.); gantsova@mail.ru (E.G.); fokichev.n@mail.ru (N.F.); makarov.olga2013@yandex.ru (O.M.); 2 Research Institute of Molecular and Cellular Medicine, People’s Friendship University of Russia (RUDN University), 117198 Moscow, Russia; 3 National Medical Research Center for Obstetrics, Gynecology and Perinatology Named after Academician V.I. Kulakov of Ministry of Health of Russian Federation, 117513 Moscow, Russia; vovankirillovmbf@yandex.ru

**Keywords:** colitis-associated colorectal cancer, inflammation, immunity cells, cytokines, hypoxia tolerance, gene expression

## Abstract

One of the factors contributing to colorectal cancer (CRC) development is inflammation, which is mostly hypoxia-associated. This study aimed to characterize the morphological and molecular biological features of colon tumors in mice that were tolerant and susceptible to hypoxia based on colitis-associated CRC (CAC). Hypoxia tolerance was assessed through a gasping time evaluation in a decompression chamber. One month later, the animals were experimentally modeled for colitis-associated CRC by intraperitoneal azoxymethane administration and three dextran sulfate sodium consumption cycles. The incidence of tumor development in the distal colon in the susceptible to hypoxia mice was two times higher and all tumors (100%) were represented by adenocarcinomas, while in the tolerant mice, only 14% were adenocarcinomas and 86% were glandular intraepithelial neoplasia. The tumor area assessed on serially stepped sections was statistically significantly higher in the susceptible animals. The number of macrophages, CD3−CD19+, CD3+CD4+, and NK cells in tumors did not differ between animals; however, the number of CD3+CD8+ and vimentin+ cells was higher in the susceptible mice. Changes in the expression of genes regulating the response to hypoxia, inflammation, cell cycle, apoptosis, and epithelial barrier functioning in tumors and the peritumoral area depended on the initial mouse’s hypoxia tolerance, which should be taken into account for new CAC diagnostics and treatment approaches development.

## 1. Introduction

Colorectal cancer (CRC) is a widespread tumor disease worldwide; its incidence is rapidly increasing annually [1]. According to GLOBOCAN, there were 19.3 million new cases and 10 million adverse cancer outcomes in 2020, with CRC accounting for 1.93 million (10%) cases and 0.94 million (9.4%) deaths, respectively. The three main types of CRC include sporadic, hereditary, and associated with inflammatory bowel disease (IBD) [2]. The epidemiological, clinical, and pathological features of IBD-associated cancers differ from sporadic and hereditary CRC types [3]. Modern diagnostic methods include not only endoscopic and histological investigations but also the immunohistochemical determination of tissue markers (glycoprotein A33, cadherin-17, and cytokeratins 7, 15, 18, and 20). Despite advances in surgery and the development of various anticancer drugs, a significant number of CRC cases are fatal.

Chronic inflammation is a determining factor in colitis-associated CRC (CAC) development, and the duration of the disease and its severity directly contribute to the risk of cancer development [4]. Patients suffering from IBD are 2–6 times more likely to develop CRC in comparison with the general population [5]. Chronic inflammation can progress via pronounced tissue regeneration and thereby intensify the initiated tumor cells’ promotion and progression [6]. The activation of nuclear factor-κB (NF-κB) and associated signaling pathways and cytokines, such as TNFα, IL-6, and IL-1β, plays a key role in the development of CAC, which leads to imbalances in cell proliferation and differentiation and tumor transformation initiation [7,8,9,10].

In the development of IBD and CAC, one of the key roles is played by hypoxia and the associated activation of the transcription factor HIF (Hypoxia-Inducible Factor), which is connected with NF-κB [11,12,13,14,15]. The *HIF1A* gene promoter has an NF-κB binding site, which, in inflammatory diseases, leads to both genes regulating the response to hypoxia and genes encoding the activation of pro-inflammatory molecules’ expressions [16,17,18,19,20]. The cellular response to oxygen deficiency is realized through the activation of the transcription factor HIF [21]. HIF is a heterodimer consisting of an HIF-α subunit (oxygen-regulated isoforms HIF-1α, HIF-2α, or HIF-3α) and a constitutively expressed HIF-1β subunit (ARNT (Aryl Hydrocarbon Nuclear Receptor Translocator)) [22,23]. It is known that tumor tissue demonstrates particular features of glucose metabolism—this is characterized by the Warburg effect, which ‘switches’ oxidative phosphorylation to glycolysis, promoting rapid energy production and the proliferation of tumor cells [24]. The active proliferation of tumor cells is followed by hypoxia and HIF-1 activation, which promotes the genes regulating key processes in tumor development and transcription, such as metabolism, angiogenesis, extracellular matrix remodeling, apoptosis/autophagy, cell survival and invasion/migration, etc. [25].

As the tumor progresses and becomes 400 μm in size, a hypoxic environment is created in its center since oxygen and nutrients can diffuse from the blood into tissues at a size of only 200 μm [26,27,28,29]. In this regard, the peritumoral area, consisting of cells in the stage of tumor transformation under acute hypoxia conditions, can be called a ‘transition zone’ between healthy tissue with sufficient oxygen supply and the tumor itself, which is under the conditions of chronic hypoxia [30,31,32]. Consequently, the cell bioenergetics in these zones differ and depend on the duration of the state of oxygen deficiency. It was also demonstrated that in tissues adjacent to the tumor, in comparison with normal autopsy samples, various cellular processes are activated (organization of vesicles and the transition from the endoplasmic reticulum to the Golgi apparatus, movement of mitochondria, cellular respiration, and the catabolism of organic molecules) and suppressed (apoptosis signaling, growth, and cell cycle) [33]. In addition, transcriptomes of peritumoral area tissues turned out to be more informative in constructing prognostic models of relapse in patients with CRC than changes in gene expression that occurred directly in the tumor [34].

The expression of certain genes in tumor diseases is different; therefore, its determination is one of the methods used to diagnose, predict, and evaluate treatment in clinical conditions. The most accurate method for such an analysis is single-cell sequencing since this approach can be used to evaluate the transcriptome of each cell separately and divide populations into tumor and normal cells. However, this method requires a developed technical base and is restricted by a limited number of studies, which makes it difficult to use in clinical practice, where work is aimed at obtaining advanced results quickly [35]. A large number of other technological approaches have been developed for gene expression analysis [36], one of which is the real-time PCR used in this work. Its advantages include reproducibility, relative simplicity, low cost, and high speed of data analysis, which make it one of the main methods used in clinical practice.

It was found that HIF-1α plays an important role in IBD pathogenesis, influencing the innate and adaptive mucous membranes’ immunity responses through NF-κB [37]. In addition, HIF-1α regulates intestinal epithelial barrier functioning, making it a potential therapeutic target for IBD-associated epithelial barrier dysfunction [38]. HIF-1α activation in CRC was demonstrated to play a key role in tumor progression and can be used as a prognostic biomarker for the unfavorable course of the disease [12,39,40,41]. According to immunohistochemical studies, HIF-1α (mRNA and/or protein) is detected in tumor cells of colon adenomas and adenocarcinomas, with more pronounced expression observed in adenocarcinomas [42,43,44]. Thus, in 66.7% of CRC cases and only in 12.25% of colorectal adenoma cases, a positive reaction to the HIF-1α protein was observed in an immunohistochemical study; it was predominantly detected in the cytoplasm of cells localized around the necrosis areas and in the areas of angiogenesis. Furthermore, HIF-1α expression was significantly higher in patients with CRC stage III in comparison with stages I–II of the disease [45]. HIF-1α inhibition is a promising strategy for the treatment of various tumor types, including CRC, which is based on the delay in angiogenesis and the decrease in cell viability under the conditions of hypoxia and inflammation [46,47,48].

It is known that organisms with different hypoxia tolerance vary according to many parameters, in particular, HIF-1 expression, the level of erythropoietin, corticosterone, and norepinephrine, the activity of antioxidant enzymes, heat shock proteins, etc. [49,50,51,52,53]. Differences in HIF-1 expression may cause different susceptibility to the development of local and systemic inflammatory and tumor processes because HIF-1 can perform both pro-inflammatory and anti-inflammatory functions. In previous studies, we established that in response to the administration of lipopolysaccharide, rats susceptible to hypoxia (SH), in comparison with those tolerant to hypoxia (TH), demonstrate a more pronounced systemic inflammatory response [54]. In addition, animals with unequal hypoxia tolerance differ in the severity of acute and chronic ulcerative colitis, induced by dextran sodium sulfate (DSS)—in SH mice, both acute and chronic colitis are more severe [55,56]. Moreover, it is known that populations living in oxygen deficiency conditions in the highlands have reduced morbidity and mortality from certain types of malignant tumors, in particular, lung, breast, and esophageal cancer [57,58]. On the contrary, a high incidence of gastric cancer is observed in the highland regions of Spain, Iran, China, and Latin America [59,60] and CRC in Ecuador [60]. Chronic hypoxia is a well-known factor for paraganglioma development among the natives residing in high altitudes. Carotid body paraganglioma incidence rises linearly with an increase in altitude and elevation, and natives of Quito, Mexico City, and the Peruvian and Bolivian Andes are at high risk of such tumor development [61].

The differences in HIF expression associated with tolerance to hypoxia can determine not only the severity of colitis but also CAC initiation and progression. Current experimental models of CRC include spontaneous, chemically induced, genetically engineered, and transplanted [62,63]. Azoxymethane (AOM) and DSS are used to model CAC that develops against the background of IBD. AOM is a carcinogen that, after metabolic activation in the organism, leads to the O_6_- or N_7_-methylation of guanine in DNA, resulting in mutagenic changes in cells [64]. In combination with DSS, AOM induced the development of adenomas (90% of cases) and adenocarcinomas (10% of cases) in experimental animals [65], which corresponds to colitis-associated neoplasia in humans. Despite the widespread use of AOM/DSS-induced CAC in experimental studies, this model is not standardized. Tumor modeling is carried out on different strains of mice that vary in age and sex, and the concentration of AOM and DSS varies. In studies on the experimental model of CAC induced by AOM and DSS, as a rule, there are no detailed pathomorphological characteristics of the developed tumors.

Therefore, this study aimed to characterize the morphological and molecular biological features of colon tumors in tolerant and susceptible to hypoxia male C57Bl/6 mice using experimental colitis-associated CRC.

## 2. Results

### 2.1. Tumor Development Frequency in Mice Tolerant and Susceptible to Hypoxia after AOM Administration and Three Cycles of DSS Consumption

**Figure 1:**
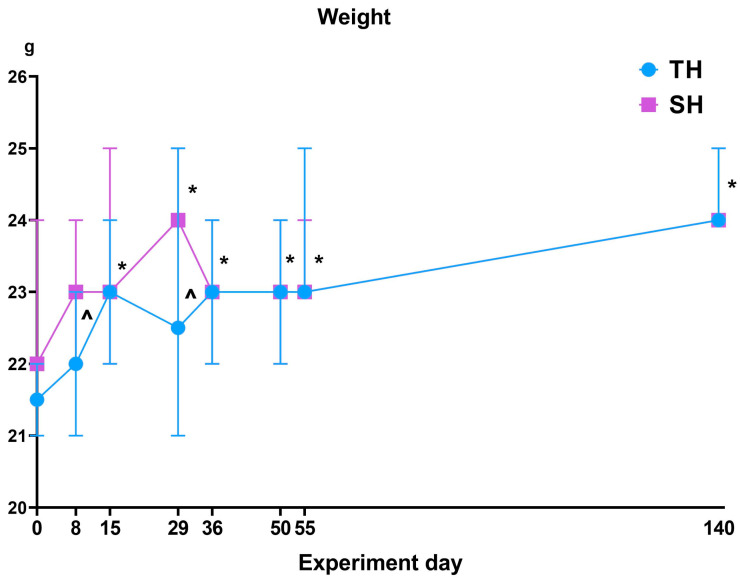
Body weight dynamics in TH and SH mice during the experiment. *—statistically significant differences relative to the beginning of the experiment; ^—statistically significant differences between TH and SH.

During clinical observation of the animals that received a single dose of AOM and three cycles of DSS, both the TH and SH mice experienced diarrhea and the presence of blood in the feces, which is associated with the development of ulcerative colitis. Furthermore, from day 15 to the end of the experiment, a change in body weight was observed in both the TH and SH mice of the experimental group in comparison with the beginning of the experiment. In addition, on days 8 and 29, the weight of the SH mice was statistically significantly higher compared with the TH group (Figure 1).

**Figure 2:**
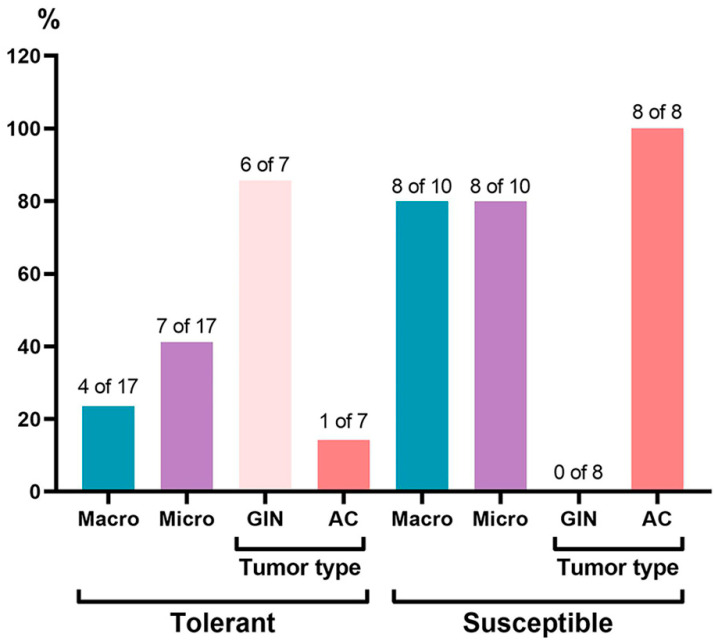
Frequency of tumor development in the distal colon of mice that were tolerant and susceptible to hypoxia on the 141st day of the experiment. GIN—glandular intraepithelial neoplasia, AC—adenocarcinoma.

Macroscopic examination in the distal colon revealed tumors in 24% (4 out of 17) of the TH animals and in 80% (8 out of 10) of the SH animals (Figure 2).

Histological examination in all three colon parts revealed chronic colitis. Single epithelialized ulcers were identified in the mucous membrane, the crypts were deformed, their lumens were enlarged, and crypt abscesses were detected. Inflammatory infiltration of lymphocytes, macrophages, and plasma cells was observed, mostly pronounced in the basal part of the mucous membrane lamina propria. At the same time, upon microscopic examination in the distal colon, tumors were detected in 41% (7 out of 17) of the TH mice and in 80% (8 out of 10) of the SH mice; in the medial colon, tumors were detected in 12% (2 out of 17) of the TH mice and in 10% (1 out of 10) of the SH mice; and no tumors were detected in the proximal colon of either the TH and SH animals. For the morphometric tumor study, PCR, and flow cytometry analysis, we used data obtained only from animals with confirmed tumors, i.e., with glandular intraepithelial neoplasia (GIN) and adenocarcinomas of the mice that were tolerant (*n* = 7) and susceptible (*n* = 8) to hypoxia.

### 2.2. Morphological Study of the Distal Colon in Mice Tolerant and Susceptible to Hypoxia after AOM Administration and Consumption of Three DSS Cycles

In the distal colon, tumors were represented by GIN, as well as adenocarcinomas, and the frequency of their development was higher in the SH mice (Table 1).

**Figure 3:**
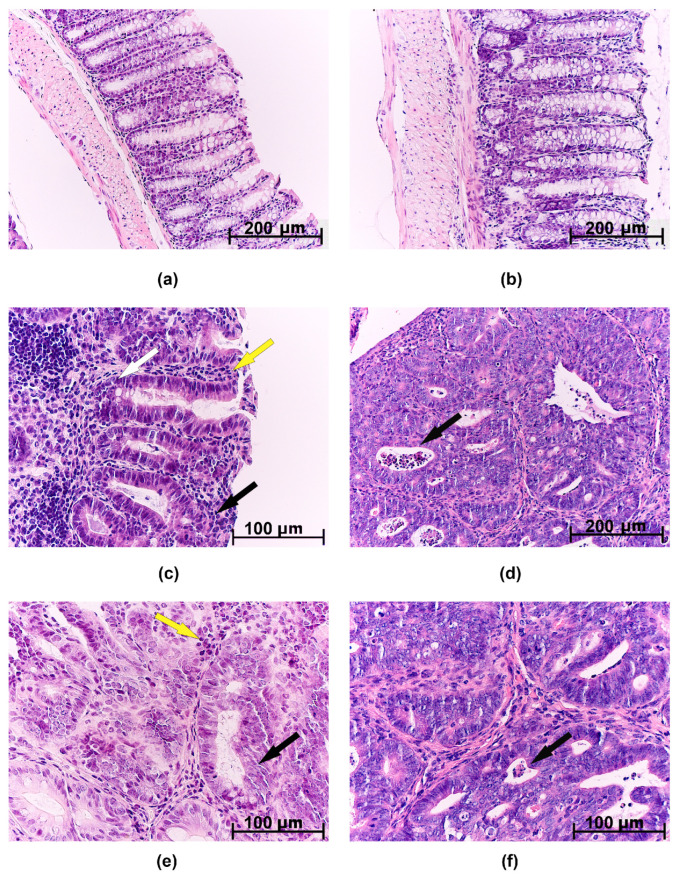
Morphological study of the distal colon in mice from experimental groups that were tolerant and susceptible to hypoxia. Control: (**a**) tolerant to hypoxia; (**b**) susceptible to hypoxia; GIN: (**c**) tolerant to hypoxia; adenocarcinoma: (**e**) tolerant to hypoxia; (**d**,**f**) susceptible to hypoxia. Hematoxylin and eosin staining. (**a**,**b**) The epithelium is preserved, crypts are high and narrow, many goblet cells, and narrow submucosa; (**c**) the yellow arrow indicates the inflammatory infiltrate, the white arrow indicates a decrease in the number of goblet cells, and the black arrow indicates disruption of crypt architectonics; (**d**,**f**) the black arrow indicates crypt abscesses; (**e**) the yellow arrow indicates atypical cells and the black arrow indicates disruption of the crypt architectonics.

On microscopic examination, GIN was characterized by clusters of several crypts with dilated and deformed lumens, lined with hyperchromic polymorphic epithelial cells (Figure 3c). All GIN foci were located within the lamina propria of the mucous membrane. In the medial colon part, GIN was detected in two TH animals, and adenocarcinoma was revealed in one SH mouse. No tumors were detected in the proximal colon of either the tolerant or susceptible to hypoxia animals.

Adenocarcinomas were represented by tumors with exophytic growth (Figure 3d). Tumor growth into the submucosa was not detected. Microscopically, tumors were represented by many glands lined with proliferating atypical columnar epithelium; in some crypts, crypt abscesses were revealed—the lumens of the crypts were expanded and filled with mucus with a large number of neutrophils. The tumor stroma was represented by connective tissue with partially ordered thin fibers; diffuse infiltration of the connective tissue was noted with a small number of lymphocytes, macrophages, and focal neutrophils (Figure 3d–f).

Based on the morphometric study, the tumor area in the distal colon of the SH animals was statistically significantly higher (*p* = 0.006) compared with the TH animals (Table 1).

### 2.3. Flow Cytometry

**Figure 4:**
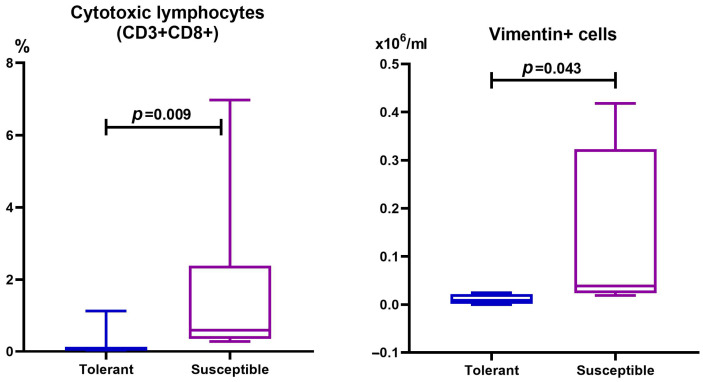
Relative number of cytotoxic T-lymphocytes and absolute number of vimentin-positive cells in tumors localized in the distal colon of mice that were tolerant and susceptible to hypoxia in CAC. Me (25–75%). *p*—statistical significance of differences, Mann–Whitney test.

In the population of cells isolated from distal colon tumors, the relative and absolute numbers of CD3+CD8+ lymphocytes and vimentin+ cells in the SH mice were statistically significantly higher than in the TH mice (Figure 4).

There were no statistically confirmed differences in the absolute and relative numbers of CD3+CD4+, CD3−CD19+, CD4+CD25+Foxp3+, NK-1.1+, or F4/80+ cells in the tumors of animals with different hypoxia tolerance.

### 2.4. HIF-1α and CRP Protein Levels in Blood Serum

**Figure 5:**
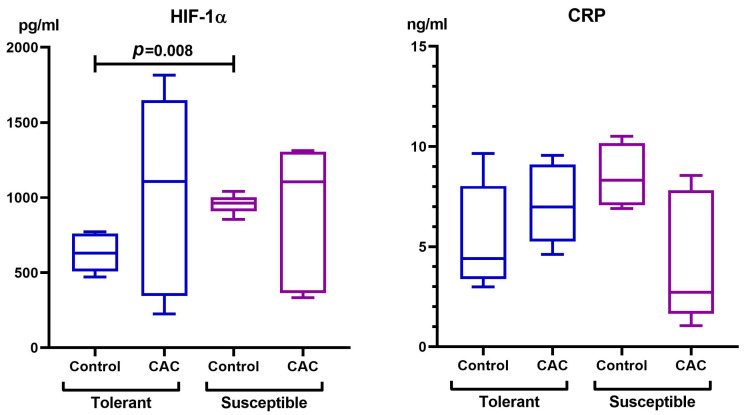
Serum HIF-1α and CRP levels in mice that were tolerant and susceptible to hypoxia in CAC. Me (25–75%). *p*—statistical significance of differences, Mann–Whitney test.

According to the obtained ELISA results, the HIF-1α level in the blood serum of the control group of SH mice was higher in comparison with the TH animals. However, no differences were revealed in the experimental groups (Figure 5). There were no differences in the CRP level between the control and experimental groups or between the TH and SH animals (Figure 5).

### 2.5. Gene Expression Levels in the Distal Colon of Tolerant and Susceptible to Hypoxia Mice in the Control and Experimental Groups

#### 2.5.1. Genes Regulating the Response to Hypoxia

**Figure 6:**
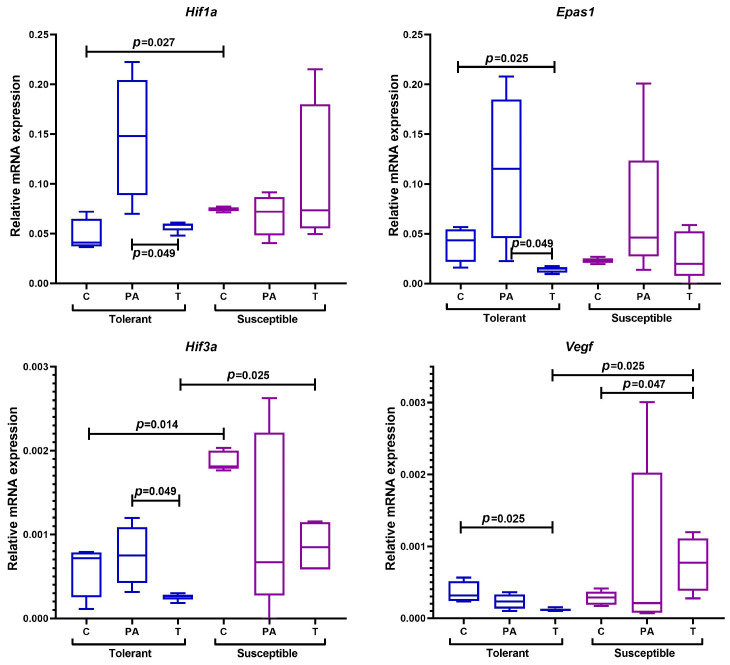
Expression levels of *Hif1a*, *Epas1*, *Hif3a*, and *Vegf* mRNA in the distal colon of mice that were tolerant and susceptible to hypoxia. Me (25–75%). *p*—statistical significance of differences, Mann–Whitney, Kruskal–Wallis, and Dunn tests. C—normal colon tissue of mice in the control groups, PA—peritumoral area tissue of mice in the experimental groups, T—tumor tissue of mice in the experimental groups.

The expression level of *Hif1a* and *Hif3a* mRNA in the distal colon in the control group was higher in the SH animals compared with the TH animals; however, the expression levels of *Epas1* and *Vegf* mRNA were not statistically significantly different (Figure 6).

Gene expression in the tumor tissue and peritumoral area was analyzed in the experimental groups of animals. The expression levels of *Epas1* and *Vegf* mRNA in the tumor tissue of the TH mice were lower in comparison with the control group, while the levels of *Hif1a*, *Epas1*, and *Hif3a* mRNA expression in the peritumoral area were statistically significantly higher relative to the tumor tissue. At the same time, *Vegf* expression increased only in the tumor tissue of the SH animals relative to the control, and no changes in *Hif1a*, *Epas1*, or *Hif3a* expression were noted either in the tumor tissues or in the peritumoral area. When comparing the expression levels of genes regulating the response to hypoxia between experimental groups with different tolerance to oxygen deficiency, it was demonstrated that *Hif3a* and *Vegf* expression in the tumors was higher in the SH mice than in the TH mice.

#### 2.5.2. Genes Regulating the Inflammatory Response

It is known that the development of CRC may be associated with IBD [66]. The CAC model used in our work is based on the combined effect of AOM, which has a mutagenic effect on cells, and DSS, which is a pro-inflammatory agent used to model acute and chronic colitis in rodents [63]. Therefore, we assessed the expression of pro- and anti-inflammatory cytokines in normal and tumor distal colon tissues, as well as in the peritumoral area.

**Figure 7:**
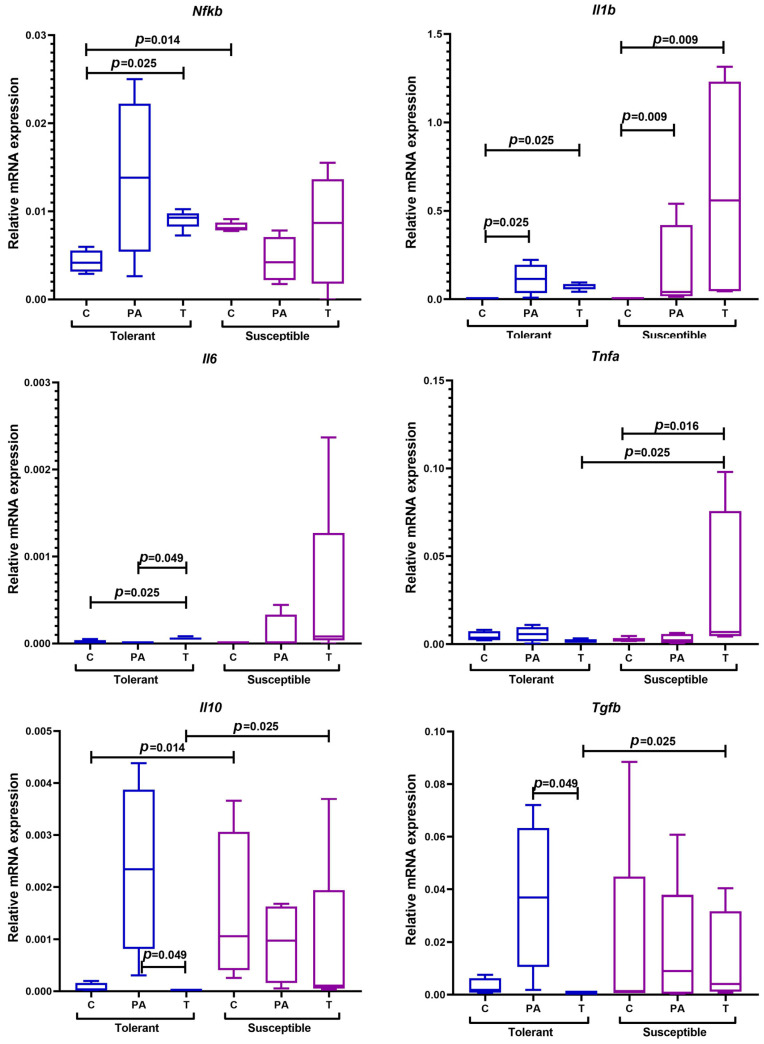
Expression levels of *Nfkb*, *Il1b*, *Il6*, *Tnfa*, *Il10*, and *Tgfb* mRNA in the distal colon of mice that were tolerant and susceptible to hypoxia. Me (25–75%). *p*—statistical significance of differences, Mann–Whitney, Kruskal–Wallis, and Dunn tests. C—normal colon tissue of mice in the control groups, PA—peritumoral area tissue of mice in the experimental groups, T—tumor tissue of mice in the experimental groups.

In the control group of animals, higher expression of *Nfkb* and *Il10* mRNA was observed in the SH mice in comparison with the TH mice (Figure 7).

In the TH animals, an increase in the expression of *Nfkb*, *Il1b*, and *Il6* in the tumors and *Il1b* in the peritumoral area was observed in comparison with the control group. Moreover, *Tgfb* and *Il10* expression in the peritumoral area was statistically significantly higher in comparison with the tumor tissue. In turn, the SH animals were characterized by high expression of *Tnfa* in tumor tissue and *Il1b* both in the tumors and in the peritumoral area compared with the control group. At the same time, in the tumor tissue of the SH mice, the expression levels of anti-inflammatory cytokines *Il10* and *Tgfb* were statistically significantly higher than in the TH mice.

#### 2.5.3. Genes Regulating the Cell Cycle

Tumor diseases, including CAC, are accompanied by phenotypic and functional heterogeneity in cells, which leads to the transformation of benign into malignant. In CRC development, there is often a sequential transformation of adenomas into adenocarcinomas caused by a neoplastic shift in the colon epithelium [67]. Thus, tumor growth is accompanied by the disruption of differentiation processes, which is associated with changes in the processes of cell growth and development. Therefore, in our experiment, we assessed the expression of cell cycle-regulating genes.

**Figure 8:**
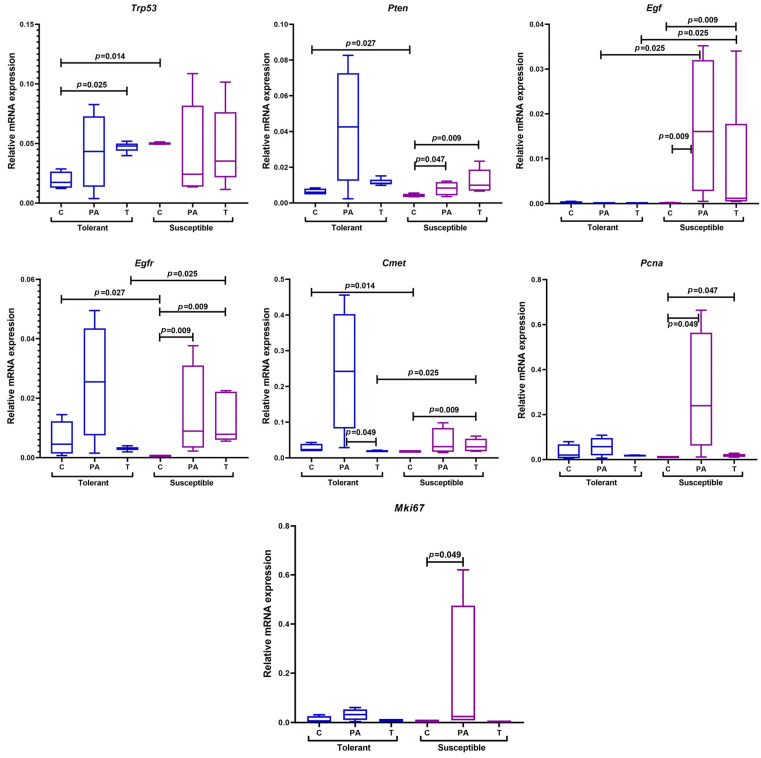
Expression levels of *Trp53*, *Pten*, *Egf*, *Egfr*, *Cmet*, *Pcna*, and *Mki67* mRNA in the distal colon of mice that were tolerant and susceptible to hypoxia. Me (25–75%). *p*—statistical significance of differences, Mann–Whitney, Kruskal–Wallis, and Dunn tests. C—normal colon tissue of mice in the control groups, PA—peritumoral area tissue of mice in the experimental groups, T—tumor tissue of mice in the experimental groups.

In the control group of SH mice, *Trp53* mRNA expression was higher and *Pten*, *Egfr,* and *Cmet* expression was reduced in comparison with the TH animals (Figure 8).

In the TH mice, *Trp53* mRNA expression in the tumor tissue was higher relative to the control mice, and the *Cmet* expression level in the peritumoral area was higher than in the tumor. In the tumor and peritumoral area of the SH animals, relative to the control animals, there was an increase in *Pten*, *Egf*, *Egfr*, and *Pcna* expression; the expression of *Cmet* increased only in the tumors, and *Mki67* increased in the peritumoral area. When conducting a comparative analysis between groups with different tolerance to hypoxia, the expression of *Egf*, *Egfr*, and *Cmet* in the tumor tissue and *Egf* in the peritumoral area in the TH animals was statistically significantly lower than in the SH animals.

#### 2.5.4. Genes Regulating Apoptosis

Mitochondrial dysfunction, leading to the inhibition of the intrinsic cell apoptosis pathway, plays a major role in tumor progression. The Bax and Bcl-2 proteins, which exhibit pro- and anti-apoptotic effects, are the main regulators of this process [68,69]. Therefore, in our study, we assessed *Bax* and *Bcl2* mRNA expression levels and calculated the *Bax*/*Bcl2* ratio.

**Figure 9:**
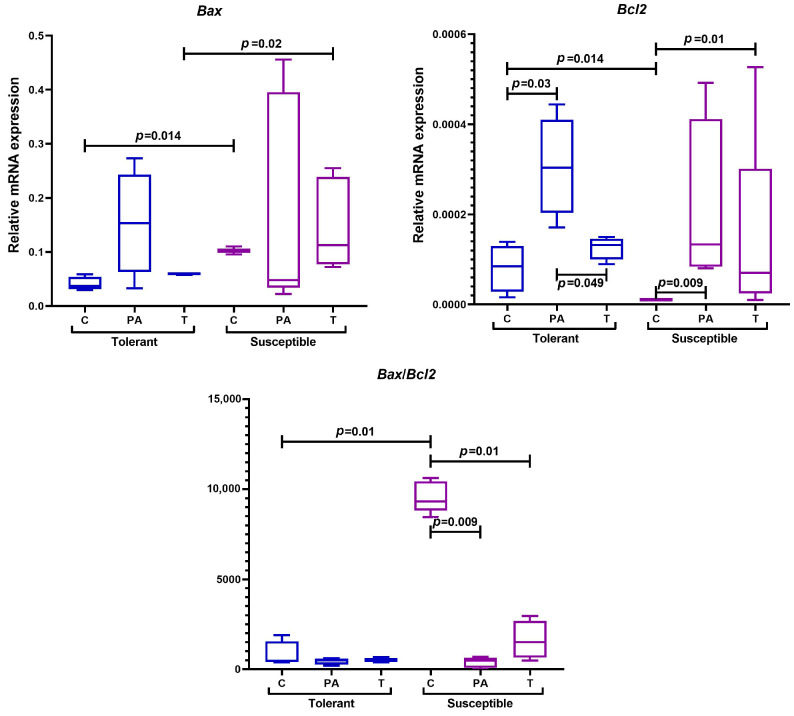
Expression levels of *Bax*, *Bcl2* mRNA, and the *Bax*/*Bcl2* ratio in the distal colon of mice that were tolerant and susceptible to hypoxia. Me (25–75%). *p*—statistical significance of differences, Mann–Whitney, Kruskal–Wallis, and Dunn tests. C—normal colon tissue of mice in the control groups, PA—peritumoral area tissue of mice in the experimental groups, T—tumor tissue of mice in the experimental groups.

In the control group, the expression of *Bcl2* mRNA in the tissues of the distal colon was higher in the TH mice, and the expression of *Bax* was higher in the SH animals (Figure 9).

In the TH mice, *Bcl2* mRNA expression increased in the peritumoral area and decreased in the tumors. At the same time, in the tumors and peritumoral area in the SH animals relative to the control animals, an increase in *Bcl2* expression was revealed. When conducting a comparative analysis between groups with different hypoxia tolerance, in the TH mice, the expression of *Bax* in tumor tissue was statistically significantly lower than in the SH animals.

An initial high *Bax/Bcl2* ratio was revealed in the SH mice of the control group. However, throughout the indicator over time assessment, after modeling CAC, a statistically significant decrease was observed only in the SH mice.

#### 2.5.5. Genes Encoding Epithelial Barrier Components

Impaired epithelial barrier function plays an important role in the development of IBD and CAC [70]. The first level of the epithelial barrier is intestinal mucus and glycocalyx, the main structural components of which are mucins [71]. By now, more than 20 genes encoding these proteins have been characterized [72]. Their main function is to protect the apical membrane from mechanical damage, which is possible because of the presence of the SEA domain in the extracellular region, the non-covalent bonds of which are characterized as quite strong [73]. Another important colon epithelial barrier component is claudins, which are proteins that form tight junctions between cells [74]. Two types of such molecules have been characterized, i.e., capping and pore-forming, but from the 27 known claudins, only 2, 10, 15, and 17 are unambiguously assigned to the second group [75]. To assess changes in the barrier function of the colon epithelium during CAC progression, we determined the levels of *Muc1*, *Muc13*, *Cldn2*, and *Cldn7* mRNA expression.

**Figure 10:**
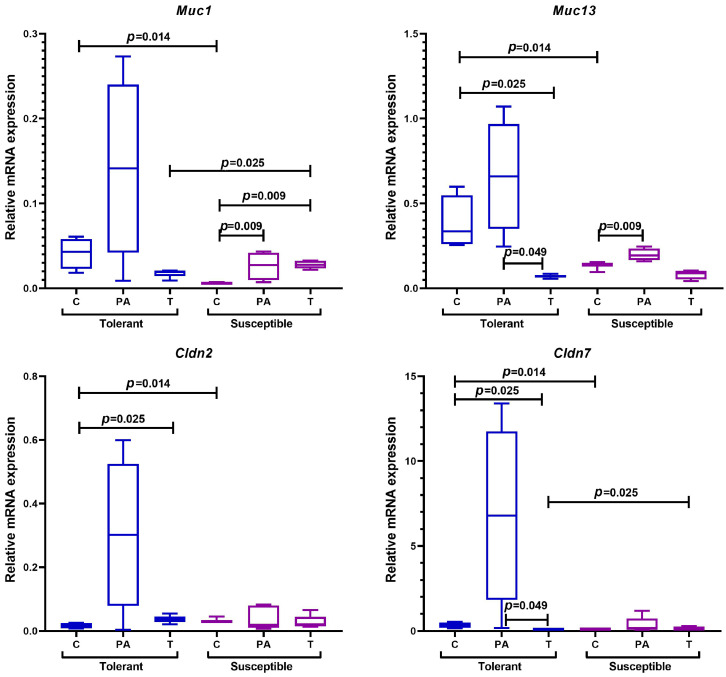
Expression levels of *Muc1*, *Muc13*, *Cldn2*, and *Cldn7* mRNA in the distal colon of mice that were tolerant and susceptible to hypoxia. Me (25–75%). *p*—statistical significance of differences, Mann–Whitney, Kruskal–Wallis, and Dunn tests. C—normal colon tissue of mice in the control groups, PA—peritumoral area tissue of mice in the experimental groups, T—tumor tissue of mice in the experimental groups.

In the control groups of animals, higher *Cldn2* expression was detected in the SH animals, while for the TH animals, higher *Muc1*, *Muc13*, and *Cldn7* expression was demonstrated (Figure 10).

In TH mice, CAC was characterized by a decrease in *Muc13* and *Cldn7* expression and an increase in *Cldn2* expression in the tumor tissue of the distal colon in comparison with the control group, while the expression of *Muc13* and *Cldn7* in the peritumoral area was higher relative to the tumors.

In turn, compared with the control group, in the tumors of the SH mice, there was a statistically significant increase in *Muc1* expression and a tendency towards a decrease in *Muc13* expression, and in the peritumoral area, there was an increase in the expression of both *Muc1* and *Muc13*. Higher *Cldn7* and *Muc1* expression was observed in the tumors of the SH mice in comparison with the THs.

**Figure 11:**
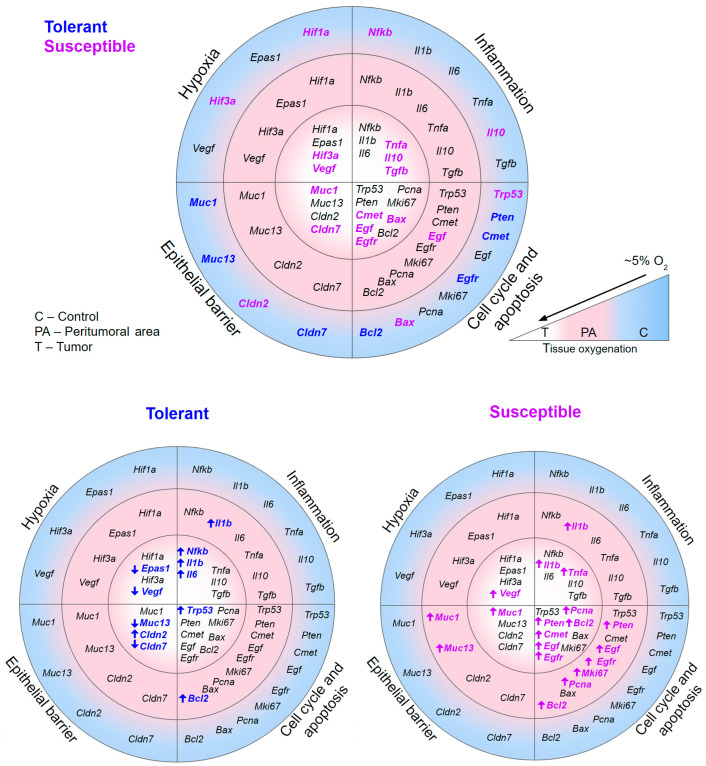
Changes in the expression of mRNA *Hif1a*, *Epas1*, *Hif3a*, *Vegf*, *Nfkb*, *Il1b*, *Il6*, *Tnfa*, *Il10*, *Tgfb*, *Trp53*, *Pten*, *Cmet*, *Egf*, *Egfr*, *Pcna*, *Mki67*, *Bax*, *Bcl2*, *Muc1*, *Muc13*, *Cldn2*, and *Cldn7* in tumors and the peritumoral area of mice that were tolerant and susceptible to hypoxia. Oxygen levels in colon tissue [76,77] are taken as normal tissue oxygen saturation. Bold blue indicates an increase in mRNA expression in the tolerant to hypoxia animals of the experimental group; bold purple indicates an increase in mRNA expression in the susceptible to hypoxia animals of the experimental group.

A summary of the results is presented in Figure 11.

## 3. Discussion

There are two CRC types caused by differences in the blood supply and embryonic origin of the colon. These include left-sided, localized in the distal third of the transverse colon, splenic flexure, descending colon, sigmoid colon, and rectum, and right-sided, in the cecum ascending colon and proximal two-thirds of the transverse colon [78]. In our work, tumors in mice were formed in the distal colon, which corresponds to left-sided localization in humans. Previously, we demonstrated that on the 71st day after AOM administration, the development frequency, tumor area, and morphological variants did not differ between TH and SH mice [79]. In this regard, the duration of the experiment was increased to 141 days, which made it possible to assess the differences in CAC progression among animals with different hypoxia tolerance. The revealed features of CAC indicate a faster tumor progression rate in the SH mice. This may be due to both the reactions of the immune system and the regulation of the various gene groups’ expression features. Impaired immune system functioning is considered an important factor contributing to the development of CRC. During CRC development, there is a significant decrease in the body’s antitumor immunity, mainly via the escape of tumor cells from immune cells [80,81,82]. The role of various types of immune cells in stimulating or inhibiting the antitumor immune response was demonstrated [83,84,85]. For instance, massive infiltration of CD8+ T cells in renal cell carcinoma is considered an unfavorable prognostic factor [86]. This probably indicates a more pronounced activation of antitumor immunity in the SH mice in response to a faster process of tumor initiation and progression.

*VIM*, a gene encoding the intermediate filament protein vimentin, is involved in maintaining cell structure and integrity and is expressed in normal mesenchymal cells. Vimentin contributes to cell shape and motility during the epithelial–mesenchymal transition (EMT) process that takes place during metastasis [87]. Vimentin was demonstrated to be highly expressed in CRC cells and plays a critical role in CRC metastasis and prognosis. The importance of vimentin in EMT is highlighted by the fact that vimentin knockdown in CRC cells reduces cell migration and invasiveness [88]. According to [89], immunohistochemical vimentin detection in 142 CRC samples demonstrated that vimentin expression in the tumor stroma is associated with poor survival. Vimentin expression in the tumor stroma reflects higher tumor malignancy potential, and high vimentin expression in CRC is associated with advanced tumor stage, metastasis, and decreased survival [89,90]. According to [90], vimentin expression does not depend on gender, age, or tumor location. However, according to the data obtained, vimentin expression differed between the TH and SH animals, which may be due to the peculiarities of its regulation depending on initial hypoxia tolerance.

According to the literature, SH animals are characterized by initially higher *Hif1a* expression and HIF-1α protein levels in comparison with TH animals [49,54], which is consistent with the data obtained. Nevertheless, information on the dependence of *Epas1* and *Hif3a* expression levels on initial hypoxia tolerance is not presented in the literature. The higher *Hif3a* expression that was first identified in the control group of the SH mice against the absence of differences in *Epas1* expression compared to the TH animals is probably due to the fact that the hypoxia resistance test is carried out in a decompression chamber under the influence of a sublethal hypoxic exposure. Since the HIF-3α isoform is active for the longest time period (48 h) after severe hypoxic exposure on cells [91], the difference we discovered between the TH and SH animals may be due to an adaptation to hypoxic conditions.

On the 141st day of the experiment, in the TH mice peritumoral area, there was probably a transition from the reaction to an acute lack of oxygen to the metabolism characteristic of chronic hypoxia (the so-called ‘HIF switch’) since normally, different HIF α-subunit isoforms are active at different time intervals of hypoxic exposure [92,93]. In addition, a significant decrease in *EPAS1* mRNA expression in tumors was observed in patients with CRC compared with normal colorectal tissue [94]. At the same time, no changes in HIF α-subunit isoform expression were observed in the SH mice, since HIF-1α, HIF-2α, and HIF-3α protein synthesis may have occurred, which activated *Vegf* expression by binding to HREs in the promoter [95], while *Hif1a*, *Epas1*, and *Hif3a* mRNA were degraded.

Higher *Hif3a* and *Vegf* expression in the tumors of the SH mice in comparison with the TH mice may indicate a later stage of tumor progression. This was confirmed via the *Hif3a* isoform, which is expressed during long-term hypoxic exposure (more than 12 h) and remains active for a long time after [91], which was typical for the tumor tissue. The high *Vegf* expression level was most likely associated with the activation of its expression by the HIF-1α and HIF-2α isoforms, which responded to oxygen deficiency in the early stages of hypoxic exposure [96]. Thus, the process of tumor growth in the SH mice occurred faster than in the TH mice.

It is known that *Nfkb* expression can be activated by hypoxic exposure [14], and the NF-kB protein has binding sites in the gene encoding the HIF-1α protein, because of which it can regulate its functioning [16]. Therefore, cellular responses to hypoxia and inflammation are interrelated. It is possible that the higher *Nfkb* expression in the SH animals of the control group was associated with a high *Hif1a* mRNA level in comparison with the TH animals. Furthermore, according to the literature, hypoxic exposure leads to changes in the intestinal microbiome [97], which can induce the development of inflammation [98]. The higher *Il10* levels in the SH animals were probably associated with the activation of the anti-inflammatory reactions neutralizing this effect. Thus, the SH animals initially had a pro-inflammatory phenotype in comparison with the TH animals.

According to the literature, in CAC, which was induced by AOM and DSS, a significant increase in IL-1β and IL-10 levels in the colon was detected [99]. It was also demonstrated that tumor growth was followed by its infiltration by macrophages that synthesize TNFα [100,101]. Furthermore, it was revealed that macrophages isolated from SH animals were characterized by a higher expression of pro-inflammatory cytokines *Il1b* and *Tnfa* compared with TH animals [101]. Thus, in this investigation, the high expression of *Tnfa* in the SH animals may be associated with the migration of macrophages into the tumor growth zone, which was confirmed by a histological examination that revealed the significant infiltration of the tumor stroma with lymphocytes and macrophages. In the TH mice, the tumor process in the colon was in its initial stages. This was confirmed by histological examination, which demonstrated that adenocarcinoma was detected in only one mouse, while GIN was detected in 6 out of 17 animals. Moreover, in the SH animals, all tumors (100%) were represented by adenocarcinomas. In the tumor tissue of the TH animals, high expression of *Nfkb*, *Il1b*, and *Il6* was observed, accompanied by high *Tgfb* and *Il10* expression in the peritumoral area. It is known that *Il1b* and *Il6* expression depends on NF-κB activity [102], and IL-6 plays an important role in the processes of invasive growth and metastasis of malignant tumors. It is capable of activating VEGF synthesis and thus influences tumor vascularization and angiogenesis [103], as well as induces IL-10 production both locally and systemically [104].

C-reactive protein (CRP) is a nonspecific marker of tissue damage during inflammation, necrosis, and various injuries [105]. Using adult male Wistar rats with different hypoxia tolerance, it was demonstrated that the CRP level increases only in SH animals 24 h after modeling a systemic inflammatory response [54]. According to the literature, CRP levels increase significantly after 10–12 h and reach a maximum at 24–48 h after the exposure [106,107,108,109,110]. It is likely that the absence of statistically significant differences between the control and the experimental groups of mice with different hypoxia tolerance in our work was due to the experiment timing.

The higher expression of anti-inflammatory cytokines genes *Il10* and *Tgfb* in the SH animals in comparison with the TH animals may be due to the negative feedback regulation to reduce high levels of pro-inflammatory molecules [111,112]. Thus, in the SH mice, tumor growth was followed by more pronounced changes in the immune system, which indicates a faster tumor progression rate. These data were also confirmed by a morphological study and the flow cytometry results—in the SH mice on the 141st day of the experiment, only adenocarcinomas were detected, while the absolute number of vimentin+ cells was also statistically significantly higher than in the TH mice.

According to the literature, p53 and PTEN are normally tumor suppressors, as well as critical factors at cell cycle checkpoints [113,114]. However, under hypoxic conditions, they make a complex, thus activating the more powerful tumor suppressor Maspin [115]. At the same time, PTEN inhibits PI3K/AKT signaling, thereby reducing the activity of the MDM2 oncogene, which is the main regulator of p53 protein transcription and degradation [116]. Thus, PTEN indirectly affects p53 functional activity. The higher *Trp53* expression in the SH mice of the control group was probably associated with the activation of regeneration processes in the intestine that are necessary for the restoration of normal function after hypoxic exposure. In turn, the low expression of *Pten* mRNA in the SH mice of the control group can be explained by the fact that the PTEN protein had already been synthesized and activated *Trp53* expression, and the observed mRNA level was residual. Simultaneously, the increased expression of *Pten* in tumors in the SH animals and *Trp53* in the TH animals was in response to the active cell proliferation [117].

EGFR is a receptor for EGF, upon binding to which signaling cascades are triggered in cells, leading to proliferation and migration [118]. It is known that colonic epithelial cells renew themselves in approximately 4–7 days [119]. It is likely that in the TH mice, as in the TH rats, reparative changes were more pronounced than in SH [120], because of the presence of more receptors on the epithelial cell surface. According to the literature, increased *EGF* expression was observed in 70–75% of established CRC cases [121], and *EGFR* was associated with various types of cancer, including CRC, breast cancer, and non-small cell lung cancer [122]. In the tumors of the SH animals, both higher *Egf* and *Egfr* expression was observed and in the peritumoral area, and a higher *Egf* expression was detected in comparison with the experimental group of TH animals, which may be associated with more active tumor cell proliferation and, accordingly, a later stage of tumor development.

High *MET* (*METamp*) expression, encoding the c-MET protein in humans, occurs in various tumor diseases. Thus, high levels of c-MET in breast cancer correlate with faster tumor growth [123], metastasis [124], tolerance to radiation therapy [125], and low overall survival [126]. Using data from the AACR Genomics Evidence Neoplasia Information Exchange (GENIE) project [127], *METamp* was demonstrated to occur in 0.4% of CRC cases (7370 patients studied) and correlated with poor prognosis [128]. In this investigation, higher *Cmet* mRNA expression in tumors was observed in the SH mice.

The Ki-67 protein is a cell proliferation marker, the expression activity of which is used in the diagnosis of various malignant diseases [129,130]. The prognostic significance of this marker remains controversial since studies by different authors demonstrated both an increase in expression and an unfavorable outcome, as well as a decrease in expression and an improved prognosis [131]. Studies conducted in the experimental model of AOM/DSS-induced CAC demonstrated that Ki-67 expression was higher in ulcerative colitis and colitis-associated dysplasia in comparison with normal tissue [132,133]. Therefore, we examined *Mki67* expression levels, as well as another proliferation marker, *Pcna*. The PCNA protein plays an important role in CRC progression and can be used as an additional marker of the malignant transformation risk of colorectal adenomas since it correlates with the dysplasia degree and tumor size [134]. According to a meta-analysis, high PCNA expression is associated with a poor prognosis and can serve as a reliable prognostic biomarker in patients with CRC [135]. Experiments on mice with AOM- and DSS-induced CAC and the mouse colon cancer cell line CT26 also demonstrated that PCNA expression is increased in tumor cells [136]. We demonstrated that the increase in *Pcna* expression was observed only in the SH mice in both the tumor tissue and peritumoral area, and *Mki67* expression increased only in the peritumoral area. At the same time, no statistically significant differences were revealed in the TH animals compared with the control group.

During CAC induced by AOM and DSS, both the TH and SH animals were characterized by an increase in *Bcl2* mRNA expression in the peritumoral area of the distal colon. According to the literature, the *Bcl2* gene product Bcl-2 protein is an anti-apoptotic factor involved in cell proliferation regulation. An increase in its expression leads to apoptosis inhibition, the emergence of tumor cells, and, as a consequence, cancer development [137]. Thus, in the peritumoral area, tumor transformation of cells most likely occurs, characterized by an increase in *Bcl2* expression relative to control groups of animals. In colon tumor tissue in the SH animals relative to the TH animals, a statistically significantly higher *Bax* mRNA expression, encoding the proapoptotic protein Bax, was observed. This difference is likely a consequence of the cellular response to mitochondrial dysfunction, which resulted in the imbalance of pro- and anti-apoptotic factors. Since there was a statistically significant increase in anti-apoptotic *Bcl2* expression in the tumors of the SH mice during CAC, it is likely that the higher pro-apoptotic *Bax* expression may be due to compensatory reactions. According to the literature, no significant correlation has been investigated between *Bax* and *Bcl2* expression and clinicopathological parameters of CRC, but the *Bax/Bcl2* ratio was correlated statistically with them [68]. It was demonstrated that a decrease in the *Bax/Bcl2* ratio is a negative prognostic marker and may indicate rapidly progressive tumor growth [68,138]. In our study, a statistically significant decrease in the *Bax/Bcl2* ratio was observed both in the peritumoral area and in tumor tissue in the SH mice, while such changes were not observed in the TH animals. Thus, in the SH animals, CAC demonstrated a more severe course and less favorable prognosis compared with the TH animals.

Hypoxic exposure can lead to the development of inflammatory reactions in an organism, including in the intestine [139]. Therefore, the hypoxia resistance test could have such an effect in animals, but at the morphological level, inflammatory changes were not identified. It is known that gene expression and cell morphology are mutually connected characteristics. Therefore, changes in morphology can influence the increase or decrease in gene expression [140], and variations in expression patterns can cause cell morphology reorganization [141,142]. However, mRNA expression and changes in morphology are also influenced by a large number of factors, including the formation of various splice variants, post-translational modifications, and protein folding disorders. In addition, changes in gene expression and cell morphology occur at different times, so one-to-one correspondence between these two modalities cannot be expected [143].

According to the literature, high *Cldn2* expression promotes healing of the intestinal mucosa and crypt regeneration [144], and *Cldn7* promotes epithelial barrier integrity [145]. It is likely that after the hypoxia resistance test in the SH mice, a more pronounced response developed in the epithelial cells of the intestine, which led to the activation of regeneration processes, while in the TH animals, this process was less pronounced. This reflects the adaptation features of the TH animals under oxygen deficiency conditions. In the tumor tissue of the TH mice, an increase in *Cldn2* expression was observed, followed by a decrease in *Cldn7* and *Muc13* expression. It was demonstrated in mice that *Cldn7* gene knockout leads to an increase in intestinal epithelium sensitivity to AOM and DSS [146], and low *Muc13* mRNA expression and high levels of MUC13 protein are IBD characteristics [147]. Also, according to the literature, mucins are normally produced by goblet cells, but via inflammation and in tumors, their numbers decrease, the secretion of proteins decreases [148], and high *Muc1* expression during tumor growth is associated with increased invasion and metastasis [149,150,151]. It is likely that the high *Cldn2* expression in the TH animals promoted intestinal epithelial tissue regeneration, which was highly sensitive to AOM and DSS, and inflammation accompanying tumor growth led to a decrease in the number of goblet cells and, as a consequence, *Muc13* expression. It is also possible that under tumor growth conditions, the energy resource of cells was directed not to the synthesis of epithelium surface structures but to the production of tight junction proteins. At the same time, *Muc1* and *Cldn7* expression was statistically significantly higher in the tumor tissue of the SH mice in comparison with the TH mice, which is likely associated with a more severe course of CAC. This was confirmed by the data from the histological study. Thus, more pronounced damage of the epithelial barrier during CAC was observed in the SH mice.

Thus, the SH animals in the experimental group were characterized by more intense tumor growth, and, perhaps, would have had an earlier natural lethal outcome, in comparison with the TH mice. According to the literature, tolerance to hypoxia correlates with overall physical fitness, toxin inactivation capacity, locomotor potential, physiological stress endurance, adaptability, and probably also immunological plasticity of the body [152]. Furthermore, TH and SH animals had differences in the structural organization and functional activity of mitochondria in cerebral cortex cells, liver, and heart [153,154]. TH animals were characterized by a high content of mitochondria with denser cristae packing and a darker matrix, a larger number of small functionally more active mitochondria, and a higher concentration of mitochondrial enzymes compared to SH animals. The demonstrated phenotypic ultrastructural, functional, and metabolic differences indicate greater respiratory chain activity in TH rats compared with SH rats. These differences suggest that energy metabolism is one of the factors determining individual hypoxia tolerance. In addition, tumor promotion and progression in TH and SH animals may depend on immune surveillance. According to our previous data, cellular and innate immunity was more pronounced in TH rats, while humoral immunity was more pronounced in SH animals [155]. It is likely that effective defense mechanisms, including antioxidant defense enzymes, heat shock proteins, and the immune response, in the TH animals contribute to slower tumor growth because of chronic inflammation.

Our study has certain limitations. Despite all the advantages of PCR, this method still has particular disadvantages. The most significant is that the material for clinical testing is usually formalin-fixed paraffin-embedded (FFPE) biopsy specimens. Although there are various commercial nucleic acid extraction kits for such samples [156], the quality of the material varies, which can be associated with massive RNA losses during extraction and tumor tissue heterogeneity [157]. In the course of this work, the research material was taken from animals and immediately transferred to fixative, which did not lead to a decrease in RNA yield at the isolation stage. Moreover, although standardized methods of biopsy and autopsy were not developed, in this work, fragments of the tumors and peritumoral areas were taken with a binocular, which is difficult to perform while working with patient material. In addition, despite the overall tumor heterogeneity, differences in gene expression between the TH and the SH mice were identified. This confirms the need to take into account the initial hypoxia tolerance of an organism when diagnosing and treating various diseases, including tumors. Therefore, in order to understand the relationship between initial hypoxia tolerance and the course of colitis-associated colorectal cancer fully, it is necessary to conduct additional studies to assess protein content in tumors and the peritumoral area by immunohistochemistry and Western blotting.

It was demonstrated that the use of AOM together with DSS promotes tumor development similar to the development, localization, and histopathological characteristics in humans [63]. Nonetheless, laboratory animals, unlike humans, are less heterogeneous because they are not exposed to various environmental changes such as diet and physical exercises. Moreover, the literature data on the pathology in human and experimental animal genetic similarity is contradictory. Thus, studying the molecular genetic features of CAC induced by AOM and DSS, including the relation to the initial hypoxia tolerance of the organisms, remains relevant for researchers.

## 4. Materials and Methods

### 4.1. Animals

This study was carried out on male C57Bl/6 mice (*n* = 70) aged 1.5–2 months, body weight 20–25 g, that were purchased from the animal breeding facility Branch of the Federal State Budgetary Institution of Science Institute of Bioorganic Chemistry named after Academicians M.M. Shemyakin and Yu.A. Ovchinnikov, Russian Academy of Sciences (FIBCh RAS), Russia. Ten mice were housed per 15 × 25 × 13 cm^3^ cage at a regulated room temperature of 25 ± 2 °C under a 12:12 h light–dark cycle and 40–50% relative humidity with unlimited access to water and food (Char, JSC Range-Agro, Turakovo, Russia). All efforts were made to decrease the suffering and possible stress of the animals. The experiments were performed in accordance with the European Convention for the Protection of Vertebrate Animals Used for Experimental Use (ETS 123, Strasbourg, 1986) and Directive 2010/63/EU of the European Parliament and of the Council of 22 September 2010 on the protection of animals used for scientific purposes. The animal study was reviewed and approved by the Bioethics Committee at the Avtsyn Research Institute of Human Morphology (Protocol No. 33(9), 7 February 2022). All procedures were in accordance with the ‘Animal Research Reporting of In Vivo Experiments’ (ARRIVE) guidelines and the AVMA euthanasia guidelines 2020. The mice were randomly divided into experimental groups with a minimum of five animals each.

### 4.2. Hypoxia Resistance Test and CAC Modeling

Hypoxia tolerance was assessed through the physiological response to oxygen deprivation using the decompression chamber test as described previously. The animals were exposed, one at a time, to simulated hypobaric hypoxia equivalent to 10,000 m altitude using a mercury barometer-coupled decompression chamber. Time length until the first sign of characteristic hyperventilatory response (‘gasping time’) was recorded using an electronic stopwatch [49,50,51,54,158,159]. Based on the results of the hypoxia resistance test, the animals were divided into 2 groups—TH (*n* = 22), whose ‘gasping time’ was more than 10 min, and SH (*n* = 15), whose ‘gasping time’ was less than 3 min. Mice that were normally tolerant (*n* = 33) to hypoxia (‘gasping time’ from 3 to 10 min) were not used in the experiments. A month after the hypoxia resistance test, the TH (*n* = 17) and SH (*n* = 10) animals in the experimental groups were modeled for CAC by a single intraperitoneal injection of AOM (A5486-25MG, Sigma-Aldrich, St. Louis, MO, USA) at a dose of 10 mg/kg [160]. A week after the AOM injection, chronic colitis was modeled in mice of the experimental groups—their drinking water was replaced with 1% DSS with a molecular weight of 40 kDa (Dextran sulfate sodium salt, Mr ~40,000, AppliChem, Darmstadt, Germany) for 7 days. After 14 days of drinking water consumption, the drinking water was replaced with 0.5% DSS for 7 days, then after another 14 days of drinking water consumption, it was replaced with 0.5% DSS for 5 days [161]. The tumor burden did not exceed the recommended dimensions (according to The University of Pennsylvania Institutional Animal Care and Use Committee (IACUC) guidelines). The animals were sacrificed after the experiment by cervical dislocation 86 days after the last replacement of drinking water with DSS in the experimental groups, that is, on the 141st day of the experiment. The TH (*n* = 5) and SH (*n* = 5) mice in the control groups were injected intraperitoneally with a physiological solution, then they consumed drinking water for 140 days.

### 4.3. Morphological and Morphometric Study

For morphological examination, the colon was divided into the proximal, medial, and distal parts, fixed in buffered 10% formalin for 24 h, and embedded in paraffin. Then, stepped-serial histological sections were prepared (from 8 to 12 sections) with a step of 200 μm and stained with hematoxylin and eosin. To assess the tumor area in the distal colon, at least 4 stepped-serial histological sections, stained with hematoxylin and eosin, in which tumors were detected were photographed using an Axioplan 2 Imaging microscope (Carl Zeiss, Oberkochen, Germany) at a magnification 50×. The tumor borders were outlined in Adobe Photoshop CS6. Next, the area of the isolated tumors was determined using the Image-Pro Plus 6.0 program, and their area was calculated for each mouse.

### 4.4. Flow Cytometry

Using flow cytometry on a Cytomics FC 500 (Beckman Coulter, Brea, CA, USA), the subpopulation composition of lymphocytes and the relative and absolute number of cells expressing vimentin in the distal colon in the experimental groups were determined. Isolation of cells from the distal colon was carried out using the method based on enzymatic treatment of tissue fragments with dispase and collagenase, followed by filtration of cells through a 100 μm and 40 μm cell strainer [162]. To analyze the cells, we used antibodies (Thermo Fisher Scientific, Waltham, MA, USA) conjugated with FITC, PE, PE-Cy5, and PE-Cy7 to CD3−CD19+ (B-lymphocytes), CD3+CD19− (T-lymphocytes), CD3+CD4+ (T-helper cells), CD3+CD8+ (cytotoxic T-lymphocytes), CD4+CD25+Foxp3+ (regulatory T-lymphocytes), NK-1.1+ (NK cells), F4/80+ (macrophages), and vimentin+ (for EMT assessment).

### 4.5. ELISA

Blood was obtained from the jugular veins and then centrifuged at 1500 rpm for 15 min. The serum was stored at −70 °C. Serum levels of CRP and HIF-1α were measured using reagent kits by Cloud-Clone Corp. (Houston, TX, USA) and FineTest (Wuhan, China), according to manufacturers’ protocols. The color reaction development was assessed in an ANTHOS 2010 microplate analyzer (Anthos Labtec Instruments, Wals, Austria).

### 4.6. Real-Time PCR

The colons were dissected at the mesentery, washed with 0.01 M PBS (pH 7.4), and opened on a Millipore filter. Under binocular control (magnification 7×), fragments of tumor tissue (areas of adenocarcinomas and areas of bright pink color, which, in a morphological study, corresponded to GIN) and peritumoral area tissue (fragments at a distance of 0.5 cm from the tumor) were isolated and fixed in IntactRNA (Evrogen, Moscow, Russia). RNA isolation and subsequent reverse transcription required for PCR were carried out using a commercial RNA Solo kit (Evrogen, Moscow, Russia) and an MMLV RT Kit (Evrogen, Moscow, Russia), respectively.

The expression of genes regulating the response to hypoxia—*Hif1a*, *Epas1*, *Hif3a*, and *Vegf*—inflammation—*Nfkb*, *Il1b*, *Il6*, *Tnfa*, *Il10*, and *Tgfb*—and the cell cycle and apoptosis—*Trp53*, *Pten*, *Cmet*, *Egf*, *Egfr*, *Pcna*, *Mki67*, and *Bax*, *Bcl2*—as well as genes encoding the epithelial barrier components—*Muc1*, *Muc13*, *Cldn2*, and *Cldn7*—in the distal colon (tumor fragments and intestinal tissue from the peritumoral area), was assessed by real-time PCR relative to the *Gapdh* expression level on a DTprime (DNA-Technology, Moscow, Russia). Primers for PCR were selected using the online program Primer3 (Biology Workbench Version 3.2; San Diego Supercomputer Center, University of California, San Diego, CA, USA) and synthesized by Evrogen (Moscow, Russia). The reaction was carried out using qPCRmix-HS SYBR (Evrogen, Moscow, Russia) with oligonucleotides in final concentrations of 0.2–0.4 μM and under thermocycling conditions described in Table S1. The relative mRNA concentration of the indicated genes was calculated by ΔΔCq [163].

### 4.7. Statistical Methods

Statistical processing of the data obtained was carried out in Statistica 8.0 and GraphPad Prism 8.0. The indicators’ distribution types were determined by the Kolmogorov–Smirnov criterion. Since the data were not normally distributed, the significance of differences among indicators was determined using the nonparametric Mann–Whitney, Kruskal–Wallis, and Dunn tests. The data were expressed as median and interquartile range Me (LQ(25%); UQ(75%)). Differences were considered statistically significant at *p* < 0.05.

## 5. Conclusions

In this study, we demonstrated for the first time that the severity of CAC induced by AOM and DSS varies in animals with different tolerance to hypoxia. The incidence of tumor development in the distal colon of the mice that were susceptible to hypoxia was 2 times higher in comparison with the tolerant mice. In addition, in the mice that were susceptible to hypoxia, all tumors (100%) were represented by adenocarcinomas, while in the mice that were tolerant, only 14% of the tumors were adenocarcinomas and the rest were glandular intraepithelial neoplasia. The average area of tumors was statistically significantly higher in animals that were susceptible to hypoxia. The content of F4/80+ macrophages, CD3−CD19+, CD3+CD4+ lymphocytes, and NK cells in tumors did not differ between animals with different hypoxia tolerance; however, the number of CD3+CD8+ cytotoxic T-lymphocytes and vimentin+ cells was higher in the mice that were susceptible to hypoxia. Changes in the expression of genes regulating the response to hypoxia—*Hif1a*, *Epas1*, *Hif3a*, and *Vegf*—inflammation—*Nfkb*, *Il1b*, *Il6*, *Tnfa*, *Il10*, and *Tgfb*—the cell cycle and apoptosis—*Trp53*, *Pten*, *Cmet*, *Egf*, *Egfr*, *Pcna*, *Mki67*, *Bax*, and *Bcl2*—as well as genes encoding the epithelial barrier components—*Muc1*, *Muc13*, *Cldn2*, and *Cldn7*—in tumors and the peritumoral area depended on the mice initial resistance to oxygen deficiency. In the control group of mice that were susceptible to hypoxia, higher levels of *Hif1a*, *Hif3a*, *Nfkb*, *Il10*, *Trp53*, *Bax*, *Bcl2*, and *Cldn2* mRNA expression were detected, while in the mice that were tolerant, there was higher expression of *Pten*, *Cmet*, *Egfr*, *Muc1*, *Muc13* and *Cldn7*. In turn, in the tumors of mice that were susceptible to hypoxia compared with the tolerant mice, higher *Hif3a*, *Vegf*, *Tnfa*, *Il10*, *Tgfb*, *Cmet*, *Egf*, *Egfr*, *Bax*, *Muc1*, and *Cldn7* expression was observed, and in the peritumoral area—only *Egf*. Thus, in the mice that were susceptible to hypoxia, changes in the expression of various gene groups throughout CAC were more pronounced, which indicates a more severe course and less favorable prognosis compared with the tolerant mice. The identified differences in tumor progression associated with individual hypoxia tolerance must be taken into account when developing new approaches to the diagnosis and treatment of CAC.

## Figures and Tables

**Table 1:** Number of mice with tumors in the distal colon, frequency of the development of GIN and adenocarcinoma (%), and the area of tumors (mm^2^) according to the morphological and morphometric studies in mice that were tolerant and susceptible to hypoxia, Me (25–75%).

Parameter	Tolerant	Susceptible
Animals with tumors	41% (seven out of seventeen)	80% (eight out of ten)
GIN	86% (six out of seven)	0% (zero out of eight)
Adenocarcinoma	14% (one out of seven)	100% (eight out of eight)
Area of tumors, mm^2^	0.44 (0.06–2.01)	3.89 (2.23–6.86)

## Data Availability

The raw data supporting the conclusions of this article will be made available by the authors upon request.

## Supplementary Materials

The following supporting information can be downloaded at: https://www.mdpi.com/article/10.3390/ijms25147801/s1.
