# Supplementary material for: Changes in the Expression of Genes Regulating the Response to Hypoxia, Inflammation, Cell Cycle, Apoptosis, and Epithelial Barrier Functioning during Colitis-Associated Colorectal Cancer Depend on Individual Hypoxia Tolerance
Source: Int J Mol Sci. 2024 Jul 16;25(14):7801. doi: 10.3390/ijms25147801 (PMC11276979; doi:10.3390/ijms25147801)
Supplement: Supplementary file 1 [file ijms-25-07801-s001.zip › ijms-3081152-supplementary.pdf]

Table S1. Nucleotide sequences for real-time PCR. Abbreviations used.

Gene	Direction	Sequence	Thermocycling conditions
Reference gene			94℃ - 300 sec<br>45 cycles: 94℃ - 10 sec<br>62℃ - 10 sec<br>72℃ - 20 sec
Gapdh	Forward	AACTTTGGCATTGTGGAAGG	
	Reverse	ACACATTGGGGGTAGGAACA	
Response to hypoxia regulating genes			
Hif1a	Forward	TGCTCTCACTCTGCTGGCTC	
	Reverse	TTTGGAGTTTCCGATGAAGG	
Epas1	Forward	CCAAACACGGAGGATATGTG	
	Reverse	GTGTGGCTTGAACAGGGATT	
Hif3a	Forward	CACAGCTCCCAATCTGCCAT	
	Reverse	GCCTGAGGTTTAATCCTTGCTAC	
Vegf	Forward	GGAAGCCGTGGTGGACACAT	
	Reverse	AAGGTAGCAGTCACCACGCA	
Inflammatory response regulating genes			
Nfkb	Forward	TTTTCGACTACGCAGTGACG	
	Reverse	CCAAGTGCAGAGGTGTCTGA	
Il1b	Forward	CCTGAACTCAACTGTGAAATGC	
	Reverse	GAAAGACACAGGTAGCTGCCA	
Il6	Forward	CCACTTCACAAGTCGGAGGC	

	Reverse	GGAGAGCATTGGAAATTGGGGT
Tnfa	Forward	GTACTCTGGCAGTGACCCCG
	Reverse	AACTGCTCCACCTTGGGCTT
Il10	Forward	TAAGTGGCAAAGGGGGCGAG
	Reverse	AGGCTGAGCCCCAATGATGT
Tgfb	Forward	GTACTCTGGCAGTGACCCCG
	Reverse	AACTGCTCCACCTTGGGCTT
Cell cycle and apoptosis regulating genes		
Trp53	Forward	TTCTCCGAAGACTGGATGACTG
	Reverse	TCCATGCAGTGAGGTGATGG
Pten	Forward	GGACCAGAGACAAAAAGGGAGT
	Reverse	CCTTTAGCTGGCAGACCACA
Egf	Forward	GCTCCGTCCGTCTTATCAGG
	Reverse	TGAGAAGTTCGGGGTCAGGA
Egfr	Forward	CCATCTGGGCCAAAGATACCA
	Reverse	GACGTAGTCCAGGAGGCAAC
Cmet	Forward	CTCCCAGCCCCTCTGCTTTC
	Reverse	ATGGGGGTTTCTGCCGTGAA
Pcna	Forward	CAAGTGGAGAGCTTGGCAATG
	Reverse	CCTCAGGACACGCTGGCAT
Mki67	Forward	AGGCGAAGTGGAGCTTCTGA

	Reverse	GCTGCTGCTTCTCCTTCACTG
Bax	Forward	GCTGATGGCAACTTCAACTG
	Reverse	CCACCCTGGTCTTGGATC
Bcl2	Forward	CATGGACTGGAGAAGGGACT
	Reverse	ACCCCATTCTTCCTGATGC
Epithelial barrier regulating genes		
Muc1	Forward	GGTGACCACTTCTGCCAACT
	Reverse	TCCTTCTGAGAGCCACCACT
Muc13	Forward	AGCATGTCCCAGCTTTCTCA
	Reverse	CCATTTGCTGCCTGAGGA
Cldn2	Forward	TGCGACACACAGCACAGGCATCAC
	Reverse	TCAGGAACCAGCGGCGAGTAG
Cldn7	Forward	GCCTTGGTAGCATGTTCCTGGA
	Reverse	GGTACGCAGCTTTGCTTTCACTG
